# Nutritional practice of pregnant women in Buno Bedele zone, Ethiopia: a community based cross-sectional study

**DOI:** 10.1186/s12978-022-01390-1

**Published:** 2022-03-31

**Authors:** Worke Sisay Yismaw, Tigist Shayi Teklu

**Affiliations:** College of Public Health and Medical Sciences, Department of Nursing, Mettu University, Mettu, Ethiopia

**Keywords:** Dietary practice, Pregnant women, Buno-Bedele

## Abstract

**Background:**

Worthy health and welfare is part of the goals set by united nation. Dietary practice is visible activities or conducts of eating habit performed by a person. Poor maternal nutrition during pregnancy were associated with higher risk of having a preterm labour, low birth-weight, Intrauterine growth restrictions and facing threats to their own wellbeing and survival.

**Objective:**

To assess the nutritional practice of pregnant women in Buno Bedele zone.

**Methods:**

A community-based cross-sectional study design was deployed to conduct this study from November 1–30, 2019 in the Buno Bedele zone, Ethiopia. The study included 592 pregnant women and a proportional sample of the size of the population was allocated to each 32 kebeles. A structured interviewer administered pretested tool was utilized for data collection. Data entry was conducted using EPI-data version 3.4 and cleaned, edited and analyzed using the SPSS version 24.0. The data were presented in the form of text, frequencies, tables and figures while logistic regression was used to discover the association between dependent and independent variables.

**Result:**

This study found that about 185 (31.2%) pregnant mothers had good dietary practice. The mothers’ educational status (AOR = 1.33, 95% CI 0.34, 2.08), income (AOR = 5.7, 95% CI, 5.1, 6.65), dietary knowledge (AOR = 3.03, 95% CI 1.98, 4.18) and pregnancy intervals (AOR = 4.16 95% CI 2.74, 6.49) were factors found to be affecting the nutritional practices of pregnant women.

**Conclusion and recommendation:**

Only 31.2% of pregnant women had good dietary practice. This indicated that the majority of study participants had a poor dietary practice, which is a concern because having poor dietary practice contributes to maternal and neonatal mortality and morbidity. To increase their nutrition practices to have a healthy pregnancy. We need to focus on; nutrition education on basic nutrients, community mobilization on dietary practices using media, work on barriers, and advocating nutrition practice activities.

## Plain Language Summary

Worthy health and welfare is part of the goals set by united nation. Dietary practice is visible activities or conducts of eating habit performed by a person. To have healthy and appropriate working of the body, eating balanced diet is very crucial. This study found that about 185 (31.2%) pregnant mothers had good dietary practice. The mothers’ educational status, income, dietary knowledge and pregnancy intervals were factors found to be affecting the nutritional practices of pregnant women. The finding indicated that the majority of study participants had a poor dietary practice, which is a concern because having poor dietary practice contributes to maternal and neonatal mortality and morbidity. To avert the problem this results healthy pregnancy. We need to focus on; nutrition education on basic nutrients, community mobilization on a diet using media, work on barriers and advocating nutrition practice.

## Introduction

Dignified health and wellbeing are among the objectives set by the United Nations. The practice of diet is a visible activity or eating behavior of a person [1]. In order to have a healthy and adequate functioning of the body, eating a balanced diet is very critical. In pregnancy, mothers need a balanced diet to support the growth and development of the fetus [2]. The nutritional condition of a mother at the time of conception, during pregnancy and breastfeeding, plays an important role in determining her health and well-being, as well as that of her newborn [3]. Malnutrition is among the most severe health problems affecting children and their mothers in Ethiopia. Malnourished mothers face higher risks during pregnancy and birth [4]. Poor maternal nutrition during pregnancy were associated with higher risk of having a preterm labour, low birth-weight, Intrauterine growth restrictions and facing with multiple threats to their own wellbeing and survival [5]. It also decreases the productivity of a woman, affecting herself, her family, community and society in general. Inadequate nutrition during pregnancy is affected by economic, geographic and cultural problems, women's information, perception and habit of eating during pregnancy, as well as inadequate nutritional intake [6].

Seven percent of the global disease burden and at least a fifth of maternal mortality are the result of maternal malnutrition along with the increased probability of poor pregnancy outcomes [7]. Poor maternal nutrition and its complications are a direct cause of newborn mortality due to premature childbirth. It is responsible for 35% (3.1 million) of global deaths in one year and indirectly raises the risk of infection-related deaths worldwide [8].

In 2011, 27% of Ethiopian women were malnourished [9] and it is one of the countries where the nutritional burden of mothers and children is high. Though, maternal under nutrition has declined over the past 16 years, from 30% in 2000 to 22% in 2016 and it is still among countries with a high burden of maternal malnutrition [10]. Regardless of a significant gains and signs of progress in the last decade, maternal nutrition still remains a major public health agenda in Ethiopia [11, 12]. The Government of Ethiopia developed a revised national nutrition program in 2016 to tackle the double burden of malnutrition among pregnant and nursing women [13, 14]. Even if its implementation requires follow-up by research, as far as our research is concerned, there is no published study in Buno-Bedele zone on this issue which helps to identify the status and factors associated with nutritional practice of pregnant women which used as an input to devise intervention.

## Methods

### Study design and setting

A community based cross sectional study was conducted in the Buno Bedele zone, which is located 480 km away from Addis Ababa. The Buno-Bedele zone is one of the zones of the Oromia region, Ethiopia. It's bordered on the south by the Illu Ababora, on the west by Kellem Wollega, on the north by Mira Wollega and on the east by Jimma zone. There are about 31 health centers and 2 hospitals in the Buno Bedele zone. According to central statistical agency's population projection, the population of the Buno Bedele zone was projected to be 707, 223 by 2017, of whom 354,168 men and 353,055 women [15]. The study was conducted from Nov. 1–30/2019 at home setting.

### Study participants

Pregnant women in the randomly selected 32 kebeles of the Buno-Bedele zone were included while those who were not able to respond due to severe illness were excluded. To select study subjects from each kebeles, systematic sampling was applied by using pregnant mothers' registration, order from health extension workers register during the data collection period. Then every 5 mothers as they registered were included in the sample until the desired sample size obtained.

### Variables of the study

Dietary practice were the dependent variable while social, demographic and socioeconomic, dietary information and gravidity, Dietary knowledge variables were the independent one.

### Sample size determination

The sample size was calculated using single population proportion formula and its determination is based on previous research finding, prevalence of good nutritional practices during pregnancy (39.9%) in Guto Gida woreda, Ethiopia [16]. Using a margin of error (0.05), 95% confidence level (Z1-ᾳ/2 = 1.96). By considering design effect & adding 10% non-response, the final sample was 610.

### Measurement

Interviewer administered structured questionnaire was used. The questionnaires designed to obtain information on socio-demographic and economic characteristics (eight questions), reproductive characteristics and factors associated with dietary practices (fifteen questions) which includes dietary knowledge and it was assessed by using 10 questions which were assumed to assess dietary knowledge of pregnant women on the aspects of nutrition required during pregnancy and to assess dietary practice of pregnant women (seven questions). Before actual data collection, two days training was given for data collectors and the supervisors about techniques of data collection and briefed on each question included in the data collection tool. The questionnaire was translated to Afan-Oromo and Amharic by language experts and then translated back to English to check for consistency with independent language experts; Pretest was done on 10% (61) of mothers in Ilubabor zone Mettu district prior to the actual study period. After pre-testing the questionnaire, Cronbatch’s alpha will be calculated to test the internal consistency (reliability) of the item. Data was collected by trained midwives and nurses and data collection regular supervision done by the supervisors. The principal investigator had trained data collectors and followed and controlled overall data collection process.

### Operational definition

*Practices* The actions of pregnant women that could affect her meal frequency per day. *Good dietary practices* pregnant women, those who scored > 75% (answered 5 questions yes or correctly) of the dietary practice question which is seven in number. *Poor Dietary practices* pregnant women, those who scored < 75% (answered 5 questions yes or correctly) of dietary practice question (Table 3) [16]. *Good dietary knowledge* pregnant women, those who scored ≥ 75% of dietary practice question. *Poor dietary knowledge* pregnant women, those who scored < 75% of dietary practice question [11].

### Data collection tool

Face to face interviews using a structured questionnaire were used for the data collection. The English version questionnaire translated into Afan Oromo and Amharic to obtain data from the study participants and to ensure clarity of its content. Then the Afan Oromo and Amharic version translated back to English version to check for consistency. The questionnaire was prepared by the investigators by reviewing different literatures [11, 16]. The questionnaires designed to obtain information on; socio-demographic and economic characteristics, obstetrics and dietary related characteristics, dietary practice, dietary knowledge of pregnant women on nutrition. The tool was pretested for its reliability and validity and we obtained 81 Cronbach alpha score.

### Data analysis

The data were checked, coded and entered using Epi data version 3.4 statistical software and analyzed using SPSS version 24.0. Data cleaning also performed to check for frequencies, accuracy, consistencies, missed values and variables. The descriptive analysis, such as percentages, means, tables, and graphs used to describe the data. Bi-variate analysis was performed to assess the association between independent variable and dependent variable which is pregnant women nutritional practice. All variables with p < 0.05 in Bivariate analysis inserted into the multivariable logistic regression model to identify factors independently associated with dietary practice of pregnant women. Significant independent predictor was declared at 95% confidence interval and P-value of less than 0.05 the cutoff point and Hosmer and Lemeshow goodness of fit test result for the final model was considered.

## Results

### Socio demographic characteristics of the respondents'

Five hundred ninety two pregnant women participated with a response rate of 97%. The mean age of the respondents was 25.34 years with ± 4.87 SD. The majority (59.6%) of the respondents were found in the age between 20 and 29 years (Table 1).

**Table 1:** Socio demographic characteristics of pregnant women in Buno Bedele zone, south west Ethiopia, Aug, 2020 (n = 592)

Variable	Category	Freq.	Percent
Age in years	15–19	88	14.9
	20–24	182	30.8
	25–29	171	28.8
	30–34	91	15.3
	35 and above	60	10.2
Religion	Orthodox	184	31.1
	Protestant	178	30.1
	Muslim	230	38.8
Educational status	Unable to read and write	118	20
	Primary education	325	54.8
	Secondary education & above	149	25.2
Marital status	Married	545	92
	Single	26	4.5
	Widowed	21	3.5
Current occupation of woman	House wife	296	50
	Farmer	236	40
	Business	24	4
	Government employee	36	6
Respondents husband occupation	Farmer	444	75
	Business	95	16
	Government employee	53	9
Ethnicity	Oromo	484	82
	Amhara	90	15
	Tigray	12	2
	Gurage	6	1
Monthly income	≤ 1000 ETB(40 USD)	176	29.7
	1000–2000 ETB(40–80 USD)	297	50.2
	≥ 2000 ETB(80 USD)	119	20.1

ETB, Ethiopian birr

### Obstetric and nutrition related characteristics of the study participants

Regarding obstetric and pregnancy related characteristics two hundred sixty eight (45.2%) were having less than or equal to two pregnancy experiences. Most of them (65.2%) were having two to five years pregnancy intervals. Two hundred thirty two (39.2%) was had three to four family size. More than half (59.1%) of them were having access to information about nutrition during pregnancy and about three hundred eighty seven (65.4%) of them were having normal nutritional status (Table 2).

**Table 2:** Obstetric and nutrition related information of pregnant women in Buno Bedele zone, south west Ethiopia, Aug, 2020 (n = 592)

Variable	Category	Freq.	Percent
Number of pregnancy	≤ 2	268	45.2
	3–5	204	34.5
	> 5	120	20.3
Parity	< 2	252	42.6
	2–5	226	38.1
	> 5	114	19.3
Pregnancy interval	< 2 years	50	8.4
	2–5 years	386	65.2
	> 5 years	156	26.4
Family size	≤ 2	197	33.3
	3–4	232	39.2
	≥ 5	163	27.5
Access to information about nutrition during pregnancy	Yes	350	59.1
	No	242	40.9

### Dietary practices of the study participants

The results of dietary practice indicated that, half (298) of them were faced craving for items not normally consumed. One hundred eighty eight (31.8%) of them were avoided certain foods in the current pregnancy, of which 30.2% avoids food due to culture. Almost half of them (317) had taken food one to two times per day (Table 3).

**Table 3:** Dietary practices of pregnant women in Buno Bedele zone, south west Ethiopia, Aug, 2020 (n = 592)

Variable	Category	Freq	Percent
Facing any cravings for items that you would normally not consume	Yes	298	50.4
	No	294	49.6
Avoiding any food or diet in the current pregnancy	Yes	188	31.8
	No	404	68.2
Reason of avoidance of any food or diet in the current pregnancy?	Religion	101	17.1
	Culture	179	30.2
	Make the baby big	137	23.2
	Makes delivery difficult	79	13.3
	Dislike and discomfort	96	16.2
Current diet frequency per day	1–2	317	53.5
	3–4	186	31.4
	> 5	89	15.1
Habits of eating snacks between meals during pregnancy	Yes	274	46.3
	No	318	53.7
Habits of eating more carbohydrate between meals during pregnancy	Yes	149	25.1
	No	443	74.9
Following your weight during pregnancy	Yes	390	65.8
	No	202	34.2
Overall dietary practices	Good	185	31.2
	Poor	407	68.8

### Factors associated with dietary practices of pregnant women

**Fig. 1:**
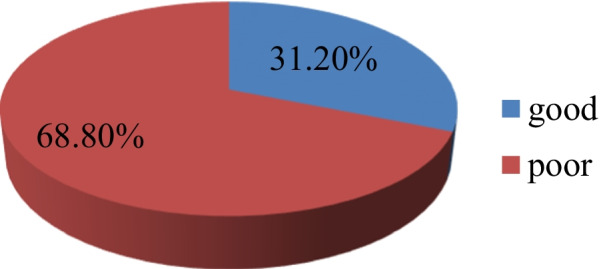
Dietary practice of pregnant women in Buno-Bedele zone, south west Ethiopia, Aug, 2020 (n = 592)

In a multivariate logistic regression analysis; Mothers educational status, income, dietary knowledge and pregnancy interval were revealed significant association with dietary practices (P < 0.05). Those study participants whose educational status is secondary education and above were 1.33 times more likely to have good dietary practice than those unable to read and write (AOR = 1.33, 95% CI 0.34, 2.08). Those study participants who earn > 2000 ETB per month were 5.7 times more likely to have good dietary practice than those earns less than or equal to 1000 ETB (AOR = 5.7, 95% CI 5.1, 6.65). The study participants who have good dietary knowledge were 3.03 times more likely to have good dietary practice than their counterparts (AOR = 3.03, 95% CI 1.98, 4.18). The study participants who had pregnancy interval of less than two years were 4.16 times more likely to have good dietary practice than those who have greater than 5 years pregnancy interval (AOR = 4.16 95% CI 2.74, 6.49) (Table 4), (Fig. 1).

**Table 4:** Factors associated with dietary practices of the pregnant women in Buno Bedele zone, south west Ethiopia, Aug, 2020 (n = 592)

Variable	Category	Dietary practice		COR (95% CI)	AOR (95% CI)	P-value
		Good	Poor			
Mothers educational status	Unable to read& write	33 (27.9)	85 (72.3)	1	1	
	Primary	115 (35.4)	210 (64.6)	1.41 (0.47, 3.04)	1.52(0.47, 3.45)	
	Secondary & above	37 (24.8)	112 (75.2)	0.85 (0.28, 1.70)	1.33 (0.34, 2.08)*	0.03
Income	≤ 1000	55 (31.3)	121 (68.7)	1	1	
	1000–2000	43 (14.5)	253 (85.5)	0.37 (0.23, 0.64)	0.36(0.23, 0.55)	
	≥ 2000	87 (72.5)	33 (27.5)	5.8 (5.17, 6.78)	5.7 (5.1, 6.65)*	0.000
Dietary knowledge	Good	135 (39.7)	205 (60.3)	2.66 (1.47, 4.27)	3.03 (1.98, 4.18)*	0.001
	Poor	50 (19.8)	202 (80.2)	1	1	
Pregnancy interval	< 2 years	30 (60)	20(40)	4.46 (3.06, 6.65)	4.16 (2.74, 6.49)*	0.01
	2–5 years	116 (30.1)	270 (69.9)	1.28 (0.80, 1.85)	1.45 (0.85, 2.32)	
	> 5 years	39 (25)	117 (75)	1	1	

^*^Statistically significantly associated

## Discussion

Proper nutrition is one of the most important factors influencing health and well-being, particularly during pregnancy. The study revealed that about 185 (31.2%) of pregnant women had good dietary practices. This suggests that only one third of them had good dietary practices, which is low. This finding is almost similar (31.4%) with studies conducted in Dale Woreda [17], Sidama zone, SNNPRS, Ethiopia and it's also almost consistent (33.9%) with studies conducted in Guto Gida woredas, East Wollega zone, Ethiopia [16].

This is higher than that of the study in Ambo Oromia District, Ethiopia [18], which was 26.9% and the study in Wando Gannet District [19], Southern Ethiopia (21.6%). The variation could be attributed to difference in; Social demography, sample size, differences in study year, improvement in dietary practices may be due to Ethiopian government is currently promoting nutrition related interventions through health extension program and active involvement of pregnant women in antenatal care as well as in one-five network meeting at community level.

The result, finding is lower than the study done in Gondar town, Ethiopia (40.1%) [20], in Northwestern Ethiopia (39.3%) [11], in America [21] more than half of women (54%) and in Malaysia (74%) [22]. The reason for the variation may be different in social, demographic, cultural, economic, sample size and cultural variability between the study settings.

This study found that mother's educational status was significantly associated with dietary practices of pregnant women (AOR = 1.33, 95% CI 0.34, 2.08). This may be due to as the mother educational increase her awareness, knowledge, access to information, attitude about nutrition practice increases by reading some materials, by following media and by attentively following her ANC care may increase. This result is in line with a study conducted in Gondar town North west, Ethiopia [20] which also supported by the study conducted in America [21] in which women with high level of education had the highest mean (%) of their nutritional knowledge and practice compared to low and moderate educational level.

The finding of this study identified that income was significantly associated with dietary practices of pregnant women (AOR = 5.7, 95% CI 5.1, 6.65). This may be as income increase their level of seeking health care specially Ante natal care increase, as income increases their capacity in using recommended food or practicing nutrition, increase and as income increase their capability in suing different information, education and communication materials may increase.This result is in line with a study conducted in Ambo district, Ethiopia [18]; it's also consistent with a study done in Gondar town north west [20] and a study conducted in Northwestern Ethiopia [11].

The finding of this study revealed that dietary knowledge was significantly associated with dietary practices of pregnant women (AOR = 3.03, 95% CI 1.98, 4.18). This may be when a woman had good dietary knowledge; she understands the issue more and practice it. Additionally, good knowledge about nutrients and balanced diet usually resulting in positive dietary practices. It’s in line with a study done in northwestern Ethiopia [11], in Gondar town [20] and in America [21].

The study also found that pregnancy interval was significantly associated with dietary practices of pregnant women (AOR = 4.16 95% CI 2.74, 6.49). This may be as the pregnancy interval increases, implementing the recommended nutrition related practice increases because those advises and health promotion activities in the previous pregnancy will be resumed since it happened in the last few years ago.

## Strengths and limitations of the study

The strength of the study was high response rate and used appropriate tools. Whereas, cross-sectional nature of the study: the study used cross sectional study design; hence it is not possible to clearly establish cause-effect relationship between the variables was the limitation.

## Conclusions and recommendation

This study found that about 185 (31.2%) of pregnant women had good dietary practice. This indicated that the majority (68.8%) of the study participants had a poor dietary practice during pregnancy, which is a concern because having poor dietary practice contributes to maternal and neonatal mortality and morbidity. We need focus on; nutrition education on basic nutrients and adequate and balanced diet, increasing household income through providing income generation activities on the behalf of the government should be created, community mobilization on dietary practices of pregnant women using media, work on the barriers, awareness creation of husbands and advocating nutrition practice activities.

## Data Availability

The datasets used and/or analyzed during the current study are available from the corresponding author on reasonable request.

## Abbreviations

ANC: Antenatal care
CSA: Central Statistics Agency
ETB: Ethiopian Birr
HSTP: Health Sector Transformation Plan
IRB: Institutional Review Board
MMR: Maternal Mortality Ratio
SBA: Skilled Birth Attendant
SDGs: Sustainable Development Goals
SPSS: Statistical Packages for Social Science
SRS: Simple random sampling and UN-United Nations

## References

[1] Assembly United General. Draft Outcome Document of the United Nations Summit for the Adoption of the Post-2015 Development Agenda. United General Assembly: New York, NY, USA; 2015.

[2] Desalegn K, Pragya S, Debebe M. J Pharm Sci Innovation.

[3] Barker DJ, Godfrey KM, Gluckman PD, Harding JE, Owens JA, Robinson JS (1993). Fetal nutrition and cardiovascular disease in adult life. Lancet.

[4] Infant and Young Child Nutrition project literature review prepared for the Message and materials development workshop produced through support provided by the United States Agency for International Development (USAID), Addis Ababa, Ethiopia; 2011.

[5] Riang’a RM, Broerse J, Nangulu AK (2017). Food beliefs and practices among the Kalenjin pregnant women in rural Uasin Gishu County, Kenya. J Ethno Biol Ethno Med.

[6] Linkages A. Maternal Nutrition: Issues and Interventions; computer based slide presentation, by the Bureau for Global Health of the United States Agency for International Development (USAID). Updated August; 2004.

[7] McGuire S, World Health Organization (2015). Comprehensive implementation plan on maternal, infant, and young child nutrition Geneva, Switzerland, 2014. Adv Nutr..

[8] Soon BT (2012). The global action report on preterm birth.

[9] Central Statistics Agency International. Ethiopia demographic and health survey 2011. Addis Ababa, Ethiopia and Calverton, Maryland, USA: Central Statistical Agency and ICF International. 2012; 430.

[10] Loudyi FM, Kassouati J, Kabiri M, Chahid N, Kharbach A, Aguenaou H (2016). Vitamin D status in Moroccan pregnant women and newborns: reports of 102 cases. Pan Afr Med J..

[11] Nana A, Zema T (2018). Dietary practices and associated factors during pregnancy in northwestern Ethiopia. BMC Pregnancy Childbirth.

[12] Gebre B, Biadgilign S, Taddese Z, Legesse T, Letebo M (2018). Determinants of malnutrition among pregnant and lactating women under humanitarian setting in Ethiopia. BMC Nutr.

[13] World Health Organization. The state of food security and nutrition in the world 2018: building climate resilience for food security and nutrition: Food and Agriculture Organization; 2018.

[14] Federal Democratic Republic of Ethiopia. National Nutrition Program 2016–2020. 2016.

[15] Central Statistics Agency of Ethiopia. Population projection of Ethiopia for all regions at woreda level from 2014– 2017. Central Statistical Agency of Ethiopia; 2013.

[16] Daba G, Beyene F, Garoma W, Fekadu H (2013). Assessment of nutritional practices of pregnant mothers on maternal nutrition and associated factors in Guto Gida Woreda, East Wollega Zone, Ethiopia. Sci Technol Arts Res J.

[17] Yoseph HH (2014). prevalence of food aversion, cravings and pica during pregnancy and their association with the nutritional status of pregnant women in Dale woreda, Sidama zone, SNNPR, Ethiopia. Int J Nutr Metab..

[18] Tolera B, Mideksa S, Dida N. Assessment of dietary practice and associated factors among pregnant mother in Ambo district, west Shoa, Oromia, Ethiopia, 2018. Ethiop J Reprod Health. 2018; 10(4).

[19] Kuche PS, Debebe M (2015). Dietary practice and associated factors among pregnant women in Wando Genet district southern Ethiopia. J Pharm Sci Innov.

[20] Alemayehu MS, Tesema EM (2015). Dietary practice and associated factors among pregnant women in Gondar Town North West, Ethiopia, 2014. Int J Nutr Food Sci.

[21] Shehab L (2012). Nutritional awareness of women during pregnancy. J Am Sci.

[22] Manaf ZA, Mei LY, Yee NS, Yin CK, Teng LW (2014). Nutritional status and nutritional knowledge of malay pregnant women in selected private hospitals in Klang Valley. J Sains Kesihatan Malaysia..

